# Establishment of a quantitative PCR system for discriminating chitinase-like proteins: catalytically inactive breast regression protein-39 and Ym1 are constitutive genes in mouse lung

**DOI:** 10.1186/1471-2199-15-23

**Published:** 2014-10-08

**Authors:** Misa Ohno, Yuta Kida, Masayoshi Sakaguchi, Yasusato Sugahara, Fumitaka Oyama

**Affiliations:** 1 Department of Applied Chemistry, Kogakuin University, Hachioji, Tokyo, Japan

**Keywords:** BRP-39, Chitinase, Chitinase-like protein, Gene expression analysis, Quantitative real-time PCR system, Ym1, Ym2

## Abstract

**Background:**

Mice and humans produce chitinase-like proteins (CLPs), which are highly homologous to chitinases but lack chitinolytic activity. Mice express primarily three CLPs, including breast regression protein-39 (BRP-39) [chitinase 3-like-1 (Chi3l1) or 38-kDa glycoprotein (gp38k)], Ym1 (Chi3l3) and Ym2 (Chi3l4). Recently, CLPs have attracted considerable attention due to their increased expression in a number of pathological conditions, including asthma, allergies, rheumatoid arthritis and malignant tumors. Although the exact functions of CLPs are largely unknown, the significance of their increased expression levels during pathophysiological states needs to be determined. The quantification of BRP-39, Ym1 and Ym2 is an important step in gaining insight into the *in vivo* regulation of the CLPs.

**Methods:**

We constructed a standard DNA for quantitative real-time PCR (qPCR) by containing three CLPs target fragments and five reference genes cDNA in a one-to-one ratio. We evaluated this system by analyzing the eight target cDNA sequences. Tissue cDNAs obtained by reverse transcription from total RNA from four embryonic stages and eight adult tissues were analyzed using the qPCR system with the standard DNA.

**Results:**

We established a qPCR system detecting CLPs and comparing their expression levels with those of five reference genes using the same scale in mouse tissues. We found that BRP-39 and Ym1 were abundant in the mouse lung, whereas Ym2 mRNA was abundant in the stomach, followed by lung. The expression levels of BRP-39 and Ym1 in the mouse lung were higher than those of two active chitinases and were comparable to glyceraldehyde-3-phosphate dehydrogenase, a housekeeping gene which is constitutively expressed in all tissues.

**Conclusion:**

Our results indicate that catalytically inactive BRP-39 and Ym1 are constitutive genes in normal mouse lung.

## Background

Chitinase-like proteins (CLPs) are structurally homologous to chitinases but lack the ability to degrade chitin [1,2]. Several CLPs have been identified in mice and humans [3-14]. Mice express primarily breast regression protein-39 (BRP-39) [chitinase 3-like-1 (Chi3l1) or 38-kDa glycoprotein (gp38k)], Ym1 (Chi3l3) and Ym2 (Chi3l4), whereas humans produce YKL-40 (CHI3L1 or human cartilage glycoprotein-39), the human homologue of BRP-39, but do not synthesize Ym1 and Ym2 [3-10].

BRP-39 and YKL-40 are glycoproteins that are secreted by various cell types, including macrophages and chondrocytes as well as tumor cells [3,10,15,16]. The amino acid sequence of mouse BRP-39 shares 73% identity with that of YKL-40 [15,16]. A recent study has shown that BRP-39 and YKL-40 are functionally equivalent [17]. Ym1 shows a high degree of sequence homology to Ym2 with 91% amino acid sequence identity, but these proteins exhibit different expression patterns [5,6,9].

Based on sequence similarities, CLPs belong to the family 18 of the glycosyl hydrolases [1,2,18,19]. Family 18 of the glycosyl hydrolases includes two catalytically active mammalian chitinases, chitotriosidase (Chit1) and acidic mammalian chitinase (AMCase) [20-23]. The conserved sequence involved in catalysis in family 18 of the chitinases is DXXDXDXE, where E is assumed to be the catalytic residue [1,2,24]. It is generally assumed that the lack of chitinase activity in CLPs is due to the mutation of crucial residues within the conserved catalytic sequence during evolution [1,2,24].

Increased levels of CLPs mRNAs and/or proteins have been noted in many inflammatory conditions [2,17,25]. BRP-39/YKL-40 levels are increased in individuals with asthma, chronic obstructive pulmonary disease (COPD), cystic fibrosis, rheumatoid arthritis, inflammatory bowel disease, alcoholic cirrhosis and different types of malignant tumors [26-36]. Ym1 is synthesized during inflammation caused by parasitic infections [37]. Ym1 and Ym2 are expressed during allergic pulmonary inflammation [5]. Thus, CLPs may play important roles in many pathophysiological conditions [38,39]. However, the contribution of these proteins to the pathophysiology of these diseases remains to be determined.

Recently we established quantitative real-time PCR (qPCR) using a single standard DNA to quantify the expression levels of chitinases and reference genes [40,41]. This method enables us to quantify and compare the expression levels of multiple genes in the same scale. Because CLPs lack chitinolytic activity and detectable functions, their biochemical properties have only been partially defined [38]. The individual quantification of BRP-39, Ym1 and Ym2 is an important step in gaining insight into the *in vivo* regulation of the CLPs.

In this study, we established qPCR system to quantify the expression of BRP-39, Ym1 and Ym2 individually and compared their expression levels to reference genes using the same scale in mouse tissues. Our study shows that the expression levels of BRP-39 and Ym1 in the mouse lung are higher than those of two active chitinases and are comparable to glyceraldehyde-3-phosphate dehydrogenase (GAPDH), a housekeeping gene which is constitutively expressed in all tissues to maintain cellular functions [42-44].

## Methods

### RNA and cDNA preparation

The qPCR assay has been designed according to the Minimum Information for Publication of Quantitative Real-Time PCR Experiments (MIQE) guidelines [45,46].

We used two types of RNA samples in this research. One is the commercially available total RNA samples pooled from 200 ~ 1,200 mice (The Mouse Total RNA Master Panel, Lot number 7120017, Clontech Laboratories). The company tested rigorously the RNA integrity. We used the total RNA samples to examine the distribution of the transcripts in various mouse tissues. Moreover, we used total RNA isolated from the lungs and stomachs of 3-month-old male mice (n = 5). All animal procedures were conducted according to the Guidelines for the Care and Use of Laboratory Animals of the RIKEN and were approved by the RIKEN Institutional Animal Care and Use Committee (Approval No. H19-2B013). C57BL/6 J mice (CLEAR Japan) were bred at the RIKEN Brain Science Institute Animal Facility. Lung and stomach tissue samples for RNA analysis were immediately frozen at -80°C. Those tissues for mRNA preparation were provided by Drs. Miyazaki and Nukina at RIKEN Brain Science Institute. Total RNA was prepared from the tissues using TRIzol Reagent (Invitrogen) according to the manufacturer’s instructions. To remove the trace amounts of contaminating genomic DNA, the samples were treated with RQ1 RNase-Free DNase (Promega) according to the manufacturer’s recommended protocol. The ratio of absorbance at 260 nm and 280 nm is used to assess the purity of DNA and RNA. The ratio of each sample was ~2.0 using a BioPhotometer Plus (Eppendorf). The concentrations of the nucleic acids were determined by measuring the absorbance at 260 nm.

The total RNA samples (3 μg) were subjected to reverse transcription using random hexamers. The reaction mixture (15 μl) contained the enzyme buffer [50 mM Tris-HCl (pH 8.3), 75 mM KCl, and 3 mM MgCl_2_], 100 ng of random hexamers (Takara Bio), 10 mM dithiothreitol, and 0.5 mM deoxynucleotide triphosphates (dNTPs). After heating the solution to 60°C for 5 min and incubating the mixture at 37°C for 5 min, 200 U of recombinant murine leukemia virus reverse transcriptase (Invitrogen) was added, and the mixture was incubated at 37°C for 45 min. The reverse transcription was terminated by heating to 95°C for 5 min.

### Selection of primer pairs for qPCR

Primers for qPCR were designed based on Primer Express Software (Applied Biosystems) and were synthesized commercially (Sigma-Genosys, Sigma-Aldrich). The PCR reactions were performed in a final volume of 13 μl containing 2 x SYBR Green Master Mix (Brilliant II SYBR Green QPCR Master Mix, Agilent), 2.7 ng of mouse cDNA or appropriate dilutions of the external standards (see below), and 2.5 pmol of the primers for the three CLPs. The PCR reactions were performed using Mx3005P QPCR System (Agilent). The PCR program was as follows: 10 min of denaturation at 95°C, 40 cycles of denaturation at 95°C for 30 sec, annealing at 55°C for 1 min and polymerization at 72°C for 1 min. Melting curves were generated after amplification. The PCR products were electrophoresed on a 10% polyacrylamide gel and analyzed using the Luminescent Image Analyzer (ImageQuant LAS 4000, GE Healthcare). The nucleotide sequences of the primers that were used for the qPCR are shown in Supplementary Information (Additional file 1: Table S1). The Chit1, AMCase, pepsinogen C, GAPDH and β-actin primers have been previously reported [40].

### Construction of the mouse Refs/CLPs standard DNA

The cDNA fragments covering the PCR-target region plus 11–137 nucleotides of the flanking regions of BRP-39, Ym1 and Ym2 were amplified from a mouse tissue cDNA mixture by PCR. The forward and reverse primers are listed in Additional file 1: Table S2. The primers contained 6-bp long EcoRI, BglII or XhoI recognition sites (shown in bold and italics) and 25-bp long sequences corresponding to the nucleotides of each CLP cDNA (Additional file 1: Table S2). These primers also contain additional 4-bp extra nucleotides (underlined) to ensure the efficient cleavage of the amplified cDNAs by the restriction enzymes (Additional file 1: Table S2). Each amplified DNA fragment contained restriction sites anchored to the PCR primer sets. These PCR products were purified using the Wizard SV Gel and PCR Clean-Up System (Promega) and then digested with the appropriate restriction enzymes, separated by 2% agarose gel electrophoresis and re-purified using the Clean-Up System. Each fragment was then ligated using T4 DNA ligase (Promega). The ligated fragments were PCR-amplified again using the forward primer (EcoRI_Ym1_Fw) and the reverse primer (Ym2_Rv) (Additional file 1: Table S2). The resulting fragments were used as the CLPs standard DNA.

**Figure 1:**
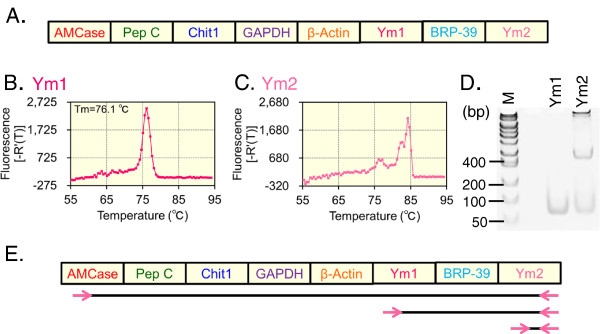
**Construction and validation of the mouse Refs/CLPs standard DNA. (A)** Schematic representation of the mouse Refs/CLPs standard DNA used for qPCR analysis. We ligated the standard DNAs of the CLPs and the reference genes (Refs) DNA [40,41] using EcoRI restriction sites and the resulting fragment was then cloned into the pGEM-T Easy vector. The linearized standard DNA was amplified from the plasmid DNA. To examine whether the standard DNA gave one melting temperature, we amplified the mouse Refs/CLPs standard DNA using the Ym1 **(B)** and Ym2 **(C)** primers. **(D)** The Ym1 and Ym2 PCR products were evaluated using 10% polyacrylamide gel analysis. **(E)** Multiple products were amplified from the mouse Refs/CLPs standard DNA using the Ym2 primers. Pink arrows indicate Ym2 primers. Lines indicate the putative amplified DNA products.

Construction of the five reference genes (Refs) and three CLPs (Refs/CLPs) standard DNA for the mouse genes was performed as previously described [40]. Briefly, mouse Refs standard DNA consisting of AMCase/pepsinogen C/Chit1/GAPDH/β-actin and containing an EcoRI restriction site at the 3’ terminus, was amplified from the standard DNA for mouse chitinase using PCR [41]. Both the CLPs and the mouse Refs standard DNAs were digested with EcoRI and ligated using T4 DNA ligase. The ligated fragments were amplified using the forward primer (Quant_mouse_AMCase_Fw) and the reverse primer (Ym2_Rv) (Additional file 1: Table S2). The PCR product was purified, and 3’-dA was added to the amplified DNA using Takara Taq HS (Takara Bio) and the resulting fragment was cloned into the pGEM-T Easy vector (Promega). The plasmid containing the cDNA insert was sequenced using the ABI PRISM Big-Dye Terminator v3.1 Cycle Sequencing Kit and the 3130 Genetic Analyzer instrument (Applied Biosystems). The mouse Refs/CLPs standard DNA (1,597 nucleotides; see Figure 1A and Additional file 1: Figure S1) was prepared by PCR reamplification from the plasmid DNA using the same primers; the PCR product was purified as described above and was thereafter used as the standard DNA.

**Figure 2:**
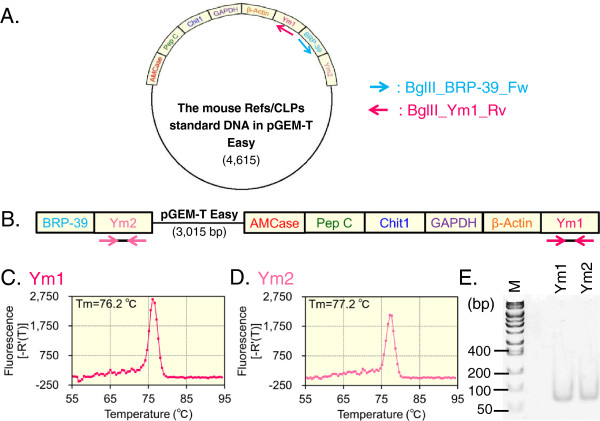
**Preparation and validation of the mouse Refs/CLPs standard DNA with pGEM-T Easy. (A)** Schematic representation of the mouse Refs/CLPs standard DNA cloning into pGEM-T Easy vector. **(B)** The mouse Refs/CLPs standard DNA with pGEM-T Easy was PCR-amplified from the plasmid DNA using the BRP-39 forward (blue arrow) and Ym1 reverse (red arrow) primers. As described above, we amplified the Ym1 and Ym2 cDNAs from this standard DNA using the Ym1 **(C)** and Ym2 **(D)** primers and these PCR products were analyzed using 10% polyacrylamide gel electrophoresis **(E)**. The y axis was expressed as first derivative of the fluorescence as a function of temperature **(C and D)**.

The mouse Refs/CLPs standard DNA with pGEM-T Easy vector (4,629 nucleotides; see Figure 2B and Additional file 1: Figure S2) was prepared by PCR from the plasmid DNA using the forward primer (BglII_BRP-39_Fw) and the reverse primer (BglII_Ym1_Rv). The amplified DNA was purified and subsequently used as the mouse Refs/CLPs standard DNA with pGEM-T Easy.

### Preparation of BRP-39, Ym1 and Ym2 cDNAs covering the entire coding region

The cDNA covering the entire coding regions of BRP-39, Ym1 and Ym2 were amplified from mouse lung (BRP-39 and Ym1) or stomach (Ym2) tissue cDNA by PCR using primers (shown in Additional file 1: Table S3) and were subcloned into the pcDNA3.1/V5-His C vector (Invitrogen). The cDNAs were sequence-verified (Additional file 1: Figure S3). The subcloned fragments were reamplified from the plasmid DNAs using the same primers (Additional file 1: Table S3), and the resulting fragments were used as the entire coding regions of the cDNAs.

### Standard curves and mRNA quantification using qPCR

The molar concentration of the standard DNA was calculated based on the concentration and the molecular weight. The concentrations of the nucleic acids were determined by measuring the absorbance at 260 nm. The molar concentration of the multigene-containing DNA standard was calculated based on the concentration and the molecular weight. Serial dilutions were prepared starting with the standard template concentration, which yielded a Ct of approximately 13 (Ct = fractional threshold cycle value). The standard DNA was subjected to 10-fold serial dilutions, ranging from 100 to 10^7^ molecules, and the aliquots were kept frozen at -20°C until use.

Standard qPCR was performed as follows: initial denaturation and polymerase activation step at 95°C for 10 min, 40 cycles of denaturation at 95°C for 30 sec, annealing at 55°C for 30 sec and polymerization at 72°C for 10 sec. The standard curves were constructed, and mRNA quantification was performed. Each sample was amplified in triplicate, and each experiment was repeated at least two times.

### Statistical analyses

Data are shown as mean with standard deviation (SD). We used Student’s t test for mRNA-level analyses. Statistical significance was set at *p* < 0.05.

## Results

### Establishment of a qPCR system for the detection of CLPs in mouse tissues

**Figure 3:**
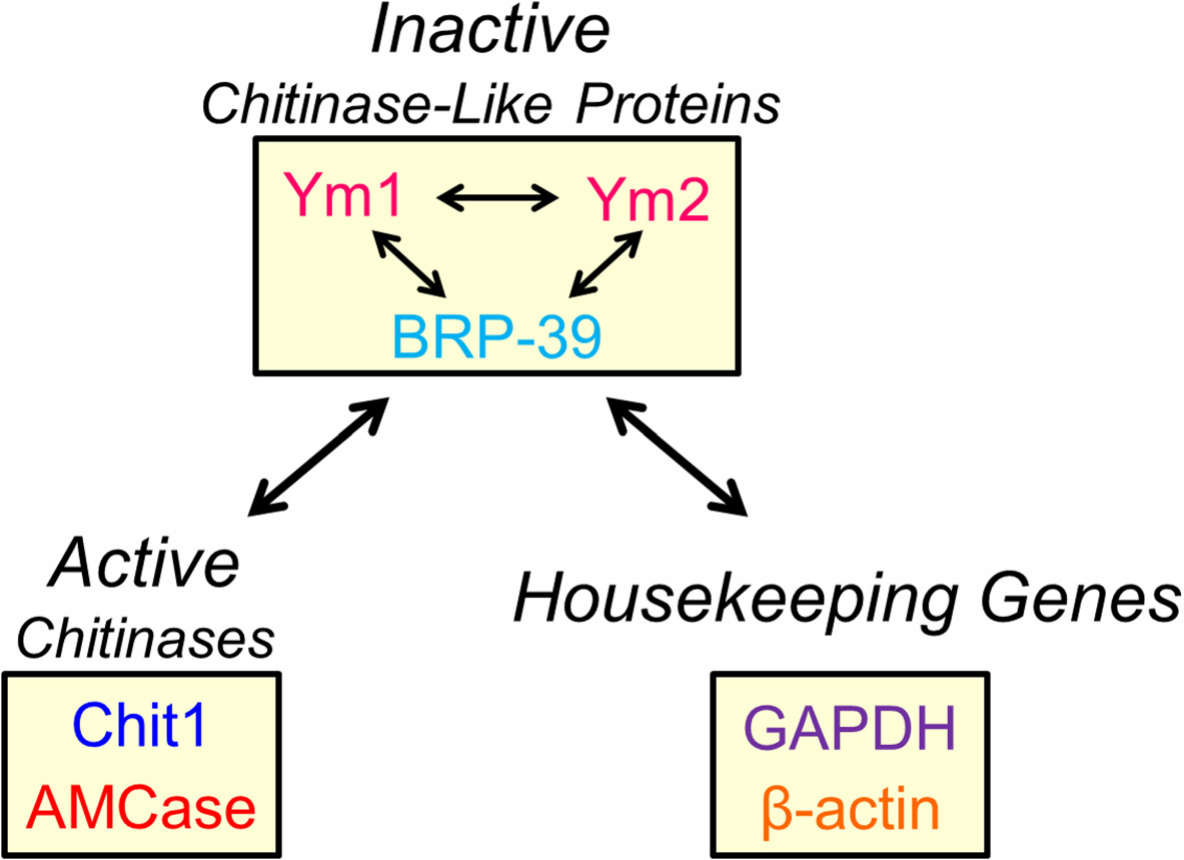
**Strategy for comparing the gene expression levels of mouse CLPs and chitinases.** The expression levels of the mouse inactive CLPs (BRP-39, Ym1 and Ym2) were quantified and compared. We then compared the expression levels of the CLPs and the active chitinases, Chit1 and AMCase, and housekeeping genes.

First, we aimed to quantify the CLPs genes expression levels across normal mouse tissues and to compare the mRNA levels of CLPs and the mammalian chitinases and reference genes (Figure 3).

**Figure 4:**
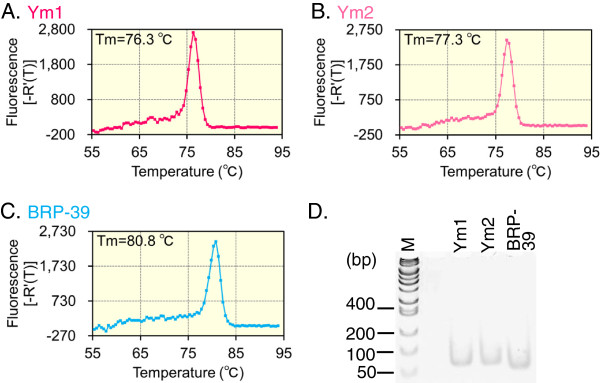
**Evaluation of the CLPs primers suitability for qPCR.** The PCR primers were evaluated based on whether they gave one melting temperature **(A-C)** and a single PCR product on a 10% polyacrylamide gel **(D)**. The y axis was expressed as first derivative of the fluorescence intensity as a function of temperature **(A-C)**. To verify specificity, the dissociation curves of the PCR products for the three genes were generated using a mouse tissue cDNA mixture. The PCR products were analyzed on a 10% polyacrylamide gel, followed by ethidium bromide staining (D).

We first established qPCR system that is capable of determining the multiple mRNAs levels using the same scale. We designed primer sets to analyze CLP expression using quantitative PCR, as described in the Methods section. Ym1 shared extensive nucleotide sequence homology with Ym2, with an identity of 94% along the entire molecule (Additional file 1: Figure S4) [9]. To prevent mispriming, we designed reverse primers that were unique at the 3’ terminal region (Additional file 1: Figure S4 and Table S1). However, because Ym1 and Ym2 share very high sequence homology, we have to choose the forward primers highly homologous between Ym1 and Ym2 (see Additional file 1: Figure S4 and Table S1). We also designed BRP-39 specific primers (Additional file 1: Table S1).We evaluated primer suitability based on whether they produced single products, as reflected by a single melting temperature (Tm) and a single band on a 10% polyacrylamide gel. The PCR products were amplified from a mouse tissue cDNA mixture. As shown in Figure 4A-C, only single peaks appeared in the dissociation curves for Ym1 (Tm = 76.3°C), Ym2 (Tm = 77.3°C) and BRP-39 (Tm = 80.8°C). Figure 4D shows clear single bands at the expected sizes for the Ym1 (65 bp), Ym2 (65 bp) and BRP-39 (57 bp) PCR fragments. These results indicate that the PCR is specific producing single amplicons from the mouse tissue cDNA mixture.

### Construction of the mouse Refs/CLPs standard DNA

We set up a qPCR system for which a standard DNA was necessary for accurate quantification of three CLPs. We used two chitinases and two housekeeping genes, GAPDH and β-actin, along with pepsinogen C, as reference genes. To evaluate the CLP levels, we used GAPDH and β-actin because they are constitutively expressed at high levels in most tissues [42-44,47]. In addition, we chose pepsinogen C (also known as progastricsin) as a reference gene in the stomach. Pepsinogen C is an aspartic protease that functions as a digestive enzyme and is produced in the stomach. This enzyme constitutes a major component of the gastric mucosa [48]. Using these reference genes, we evaluated gene expression of three CLPs and chitinases in mouse tissues.

We ligated the CLPs standard DNA with the five reference genes cDNA in a one-to-one ratio and then cloned this DNA fragment into the pGEM-T Easy vector. The 1,597-nucleotide-long DNA contained five reference genes (Refs) and three CLPs cDNA fragments that spanned the PCR target regions and 9-146 nucleotides of the flanking regions and contained several restriction sites (Figure 1A and Additional file 1: Figure S1). We name this molecule as the mouse Refs/CLPs standard DNA in this report.

### Evaluation of the mouse Refs/CLPs standard DNA with pGEM-T Easy

Next, we examined whether the target cDNAs were amplified from the mouse Refs/CLPs standard DNA using the primer sets for the eight cDNAs. Although the Ym1 primers gave a single product, as reflected by a single melting temperature and a single band on a 10% polyacrylamide gel (Figure 1B and D), the Ym2 primers yielded three peaks and multiple bands (Figure 1C and D). These results indicated that multiple products were amplified from the mouse Refs/CLPs standard DNA using the Ym2 primers.

To understand and overcome this problem, we compared the nucleotide sequences of the standard DNA and the Ym2 forward primer using the NCBI Blast Search (2 blast search) tool and found that the Ym2 forward primer can anneal to Ym1 (Additional file 1: Figure S4) and can be misprimed to the AMCase cDNAs. Ym1 and Ym2 did not share any homology with BRP-39 and Chit1. Therefore, in addition to the amplification of original Ym2 cDNA, two cDNAs (Ym1/BRP-39/Ym2 and AMCase/Pep C/Chit1/GAPDH/β-actin/Ym1/ BRP-39/Ym2) can be amplified from the standard DNA using the Ym2 primers (Figure 1C, D and E). This problem could not be solved by the annealing temperature modification.

Many researchers have designed primers that span introns or intron/exon boundaries for RT-PCR analysis [49]. We cloned the mouse Refs/CLPs standard DNA into the pGEM-T Easy vector (Figure 2A). To increase the distance between the AMCase/Ym1 and Ym2 fragments, we prepared a linear standard DNA containing the pGEM-T Easy sequence that contained BRP-39/Ym2/pGEM-T Easy/AMCase/Pep C/Chit1/GAPDH/β-actin/Ym1 by PCR using the BRP-39-forward (BglII_BRP-39_Fw) and Ym1 reverse (BglII_Ym1_Rv) primers (see Figure 2A and Additional file 1: Figure S2 and Table S2). This standard DNA is referred to as the mouse Refs/CLPs standard DNA with pGEM-T Easy (Figure 2B).

We next examined whether the Ym2 primers only amplified Ym2 cDNA from this standard DNA. To avoid the misprimed amplification, we shortened the annealing and extension time in the qPCR program (55°C for 30 sec, 72°C for 10 sec). After the PCR was performed, the Ym2 primers yielded a single product, as reflected by a single melting temperature (Figure 2D) and a single band on a 10% polyacrylamide gel at the expected size for the Ym2 PCR product (Figure 2E), as was case for the Ym1 products (Figure 2C and E); these results indicated that a single product was amplified from the mouse Refs/CLPs standard DNA with pGEM-T Easy (Figure 2B and Additional file 1: Figure S2) using the Ym2 primer. Thus, the pGEM-T Easy sequence (approximately ~3 kbp) worked as an intron sequence in the PCR reaction. Therefore, we used the mouse Refs/CLPs standard DNA with pGEM-T Easy as the standard DNA unless otherwise specified.

### Validation of the standard curve and the qPCR system

**Figure 5:**
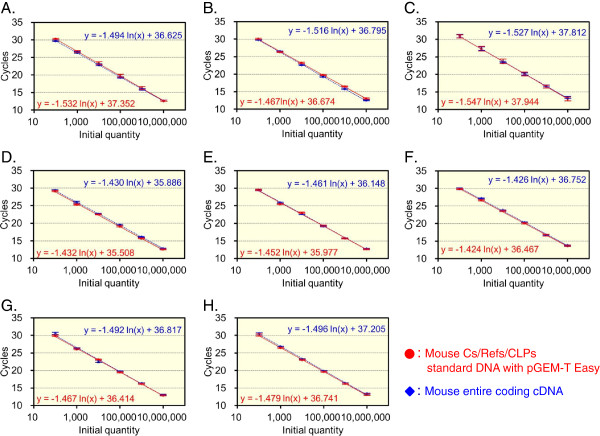
**Validation of a qPCR system for the analysis of mouse tissues.** The analyzed cDNAs were the following: **(A)**, BRP-39; **(B)**, Ym1; **(C)**, Ym2; **(D)**, Chit1; **(E)**, AMCase; **(F)**, GAPDH; **(G)**, β-actin; and **(H)**, pepsinogen C. Standard curves were obtained using the mouse Refs/CLPs standard DNA with pGEM-T Easy containing the eight mouse cDNA fragments (red closed circles). In addition, the quantification of the mouse entire coding cDNA was performed using the primer pairs for each gene. The target cDNA was amplified from a dilution of the entire coding cDNA with a known concentration and subsequently analyzed as an unknown sample (blue closed rhombuses). Equal quantities were obtained for each tested dilution of the standard curve and entire coding cDNA. Data are expressed as mean ± standard deviation (SD) of three measurements.

The quantification of the CLP, chitinase and the reference mRNAs relies on standard curves. We next examined whether the three CLPs and five reference genes mRNAs were accurately quantified using this system. Serial dilutions of the mouse Refs/CLPs standard DNA with pGEM-T Easy (Figure 2B and Additional file 1: Figure S2) were used to construct an individual standard curve to compare and evaluate the qPCR quantification strategies that were used to analyze the eight mRNAs. Each standard curve was generated using 10-fold serial dilutions of the standard DNA and the eight different primer pairs, yielding a dynamic range of seven orders of magnitude (Figure 5A-H, red closed circles).

We next evaluated the qPCR quantification by analyzing the eight cDNAs. To test the absolute equality of the curves, known concentration of the entire coding cDNA (Additional file 1: Figure S3) was amplified and subsequently analyzed as an unknown sample. As shown in Figure 5A-H, blue closed rhombuses, equal quantities were observed for each tested dilution used to construct the standard curve. Thus, we could quantify the CLPs and the reference mRNAs using the same scale.

### Expression of CLPs in normal mouse tissues

**Figure 6:**
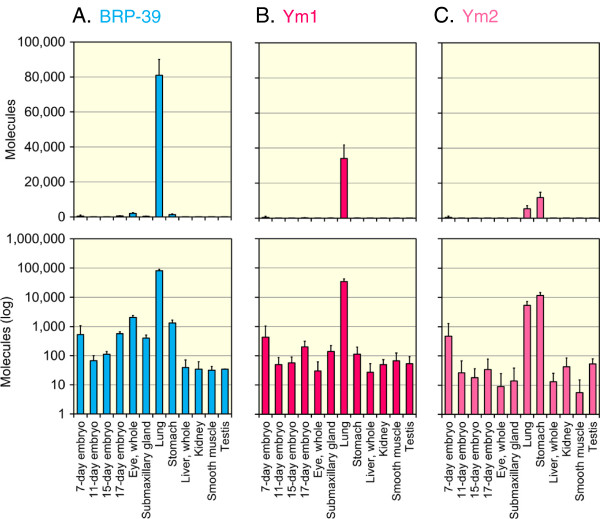
**BRP-39, Ym1 and Ym2 mRNA expression in mouse tissues.** Quantification of BRP-39 **(A)**, Ym1 **(B)** and Ym2 **(C)** mRNAs in mouse tissues. The expression levels of the CLPs were quantified by real-time PCR using the standard DNA containing the eight mouse genes (the mouse Refs/CLPs standard DNA with pGEM-T Easy). The y axis was expressed as molecules per 10 ng of total RNA. The upper panel indicates the actual value, and the lower panel shows each value on logarithmic scale. Data are presented as mean ± SD of three measurements.

To study the *in vivo* regulation of BRP-39, Ym1 and Ym2 gene expression, tissue cDNAs reverse transcribed from total RNA from four embryonic stages and eight adult tissues were analyzed using qPCR with the mouse Refs/CLPs standard DNA with pGEM-T Easy (Figure 2B and Additional file 1: Figure S2). The BRP-39, Ym1 and Ym2 mRNAs were widely expressed in the mouse tissues (Figure 6A-C).Clear tissue specificities were observed in the expression patterns of these CLPs mRNAs. BRP-39 mRNA was also widely expressed in normal mouse tissues (Figure 6A). The highest levels of BRP-39 mRNA were detected in the lung, followed by the 7-day embryo, eye, stomach and 17-day embryo (Figure 6A, upper and lower panels).Similarly, the highest levels of Ym1 mRNA were detected in the mouse lung, followed by 7-day embryo (Figure 6B, upper panel), whereas the highest levels of Ym2 mRNA were detected in the mouse stomach, followed by the lung (Figure 6C, upper panel). In other tissues, the Ym1 and Ym2 mRNAs were expressed at low, but easily detectable levels above background (Figure 6B and C, lower panel). When compared to the levels of Ym2 mRNA, BRP-39 and Ym1 were synthesized at the higher level in the lung tissue (Figure 6).

### The expression levels of CLP, chitinase and reference gene mRNAs in mouse lung and stomach tissues

Many studies on the pathophysiology of CLPs and mammalian chitinases have been performed using lung tissue [7,17,27,50-52]. In this study, we showed that Ym2 mRNA is highly expressed in mouse stomach tissue (Figure 6). Additionally, AMCase mRNA was overexpressed in mouse stomach [23,40,41,53].

**Figure 7:**
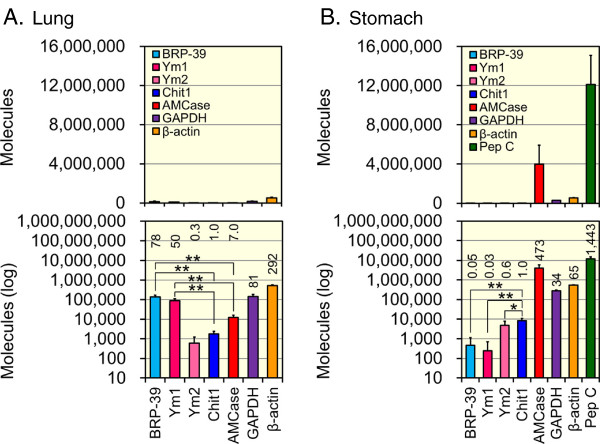
**Analysis of BRP-39, Ym1 and Ym2 reference gene mRNAs in lung and stomach tissues.** The expression levels of the eight genes determined using the cDNAs prepared from lung **(A)** or stomach **(B)** tissues from 3-month-old mice (n = 5) were quantified by real-time PCR. All of the values are expressed as number of molecules per 10 ng of total RNA in y axis. The upper panel indicates the actual value, and the lower panel shows each value on logarithmic scale. The expression level of the mouse Chit1 gene was set to 1.0; the values above the bars indicate the relative expression levels compared to the expression level of the mouse Chit1 gene. Data are presented as mean ± SD of five samples. *p < 0.05; **p < 0.01.

We next compared the expression levels of the CLPs and reference genes using the lung and stomach tissue cDNA as described in the Methods. Figure 7A shows the results obtained from mouse lung tissue. Chit1 is a well-characterized mammalian chitinase in lung tissues. When the Chit1 levels were set at 1.0, the relative expression levels of the cDNAs were 78 for BRP-39, 50 for Ym1, 0.3 for Ym2, 7.0 for AMCase, 81 for GAPDH and 292 for β-actin in the mouse lung tissues (Figure 7A). The lung tissues express higher levels of BRP-39 and Ym1 than Chit1 and AMCase, active mammalian chitinases (*p* < 0.01). Furthermore, the expression levels of BRP-39 and Ym1 in the mouse lung tissues were comparable to the level of GAPDH, a well-known housekeeping gene that is constitutively expressed at high levels in most tissues [42-44]. These results indicate that BRP-39 and Ym1 are abundantly transcribed in the mouse lung.

Figure 7B shows the results obtained in the mouse stomach tissues. When Chit1 levels were set to 1.0, the relative expression levels of the cDNAs were 0.05 for BRP-39, 0.03 for Ym1, 0.6 for Ym2, 473 for AMCase, 34 for GAPDH, 65 for β-actin and 1,443 for pepsinogen C in the mouse stomach tissues (Figure 7B). The stomach tissues express much lower levels of BRP-39 and Ym1 than Chit1 (*p* < 0.01). Although Ym2 mRNA was highly expressed in the stomach, its expression was lower than Chit1, an active mammalian chitinase (*p* < 0.05). Furthermore, the Ym2 expression level was much lower than the levels of AMCase, GAPDH, β-actin and pepsinogen C. These results indicate that Ym2 mRNA is abundant in the stomach tissues.

## Discussion

CLPs are structurally related to chitinases but lack chitinolytic activity [1,2,38]. CLPs levels are increased in a number of serious pathological conditions. Thus, the biomedical importance of CLPs has attracted considerable attention. In this study, we established a quantitation method for discriminating chitinase-like proteins and compared their mRNA levels with those of the five reference genes. We showed that catalytically inactive BRP-39 and Ym1 are constitutive genes in mouse lung.

In our previous studies, we quantified and compared Chit1 and AMCase expression levels in mouse and human tissues [40,41]. Here, we applied our methodology to the CLPs’ levels analysis. There was a concern that the primers for Ym1 and Ym2 could cross-react with each other because the nucleotide sequences of Ym1 and Ym2 are very similar. However, our primers for Ym1 and Ym2 can discriminate and amplify corresponding cDNAs from the mouse tissue cDNA mixture (Figure 4). The reverse primers for Ym1 and Ym2 contain unique sequences at their 3’ terminal regions, which strongly affect PCR efficiency, while the forward primers are similar between the Ym1 and Ym2 cDNAs (Additional file 1: Figure S4). Our results indicate that either the forward or the reverse primer can contain unique sequences for the specific amplification of a target cDNA, even though the nucleotide sequences are very similar between these cDNAs. This notion can be applied to quantify molecules that are very similar to each other, such as Ym1 and Ym2.

When we validated the mouse Refs/CLPs standard DNA, multiple products were amplified from the mouse Refs/CLPs standard DNA using the Ym2 primers. This result suggested that there could be cross-reactions between Ym1/AMCase and Ym2 (Figure 1C and E). In general, when for designing primers for RT-PCR, intron sequences are included in the target region to avoid amplifying non-target products from the contaminating genomic DNA [49]. Because mammalian introns are more than approximately ~3 kbp long, in general, genomic sequences are difficult to be amplified by PCR compared to target cDNA. Because the mouse Refs/CLPs standard DNA was cloned into the pGEM-T Easy vector (approximately ~3 kbp long, Figure 2A), we prepared a linearized mouse Refs/CLPs standard DNA with pGEM-T Easy sequence between Ym1 and Ym2 using PCR (Figure 2A and B). Additionally, we changed our qPCR protocol (annealing, 30 sec at 55°C; extension, 10 sec at 72°C). As a result, we overcame the problems, and through the validation of the mouse Refs/CLPs standard DNA with pGEM-T Easy, we could individually quantify Ym1 and Ym2 using the standard DNA (Figure 5).

Our results for the gene expression pattern of BRP-39, Ym1 and Ym2 were essentially consistent with previous reports [3,9,37]. Additionally, our analysis was sufficiently sensitive to detect the CLPs and to provide a comprehensive survey of the gene expression patterns of the CLPs and reference genes using the same scale in mouse tissues. The order of mRNA levels in mouse lung tissues were as follows: GAPDH ≈ BRP-39 ≈ Ym1 > AMCase > Chit1 > Ym2.

We found that BRP-39 and Ym1 mRNA displayed similar expression patterns (Figure 6A and B). Ym1 mRNA was expressed at high levels in mouse lung, whereas Ym2 mRNA was detected in stomach, followed by lung. Although Ym1 and Ym2 show high sequence homology, there is differential expression between them. A detailed characterization of the promoter regions of the CLPs genes and the identification of the *cis*- and *trans*-acting factors will be required to understand the selective gene expression of these CLPs in mice.

In mouse lung tissues, we found that the mRNA levels of BRP-39 and Ym1 were higher than Chit1 and AMCase, which are active chitinases. The levels of BRP-39 and Ym1 mRNA were comparable to those of GAPDH, a typical constitutive gene. In the lungs, Chit1 and AMCase can act as part of the host defense system to protect against chitin-containing pathogens, such as fungi and mites [50,54]. Compared with active chitinases, BRP-39 and Ym1 are highly expressed in the mouse lung. In addition, BRP-39 and Ym1 seem to be co-expressed in mouse lung (Figure 6A and B). Qureshi et al. reported that BRP-39, Ym1 and Ym2 are overexpressed in a model of inflammation-promoted incipient neoplasia [55]. They suggested that these CLPs may promote tissue remodeling and amplify immune responses [55]. Although BRP-39 and Ym1 have been reported to lack detectable chitinolytic activity, the high levels of their expression in mice suggest the physiological importance as biological defense in the mouse lung.

AMCase is predominantly overexpressed in the mouse stomach [40,41], and a robust peak of activity was observed at pH 2.0, suggesting that AMCase can function as a digestive enzyme that breaks down chitin-containing foods [23,56]. Chit1 is highly expressed (about 10-folds) in stomach as compared to lung tissue (Figure 7). Because Chit1 does not possess any chitinolytic activity at low gastric pH 2 [21,57], it seems that Chit1 does not contribute to chitinase activity in stomach. It has been shown that Chit1 is produced at sites of near-neutral pH, such as the non-glandular portion of the stomach and the small intestine [53]. Thus, Chit1 may also function as a digestive enzyme that breaks down polymeric chitin under the neutral tissue conditions such as small intestine.

Ym2 expression was lower than Chit1 and AMCase but was much higher compared to BRP-39 and Ym1. The function of Ym1 is not yet known, although surface plasmon resonance has demonstrated that Ym1 can bind to chitobiose, chitotriose and chitotetraose; additionally, heparin sulfate has also been suggested as a candidate ligand [8]. Ym2 is also a CLP protein of unknown function that is closely related to Ym1 [5]. The high expression of Ym2, together with AMCase, may be involved in food processing and defense mechanisms in the mouse stomach.

Increased levels of chitinase and CLP mRNAs and/or proteins have been noted in many inflammatory conditions [2,17,25]. The level of Chit1 is elevated in Gaucher disease, in smokers and in patients with chronic obstructive pulmonary disease (COPD) and Alzheimer disease [20,52,58,59]. AMCase expression and activity are also up-regulated during allergic airway responses in mouse models of asthma and by polymeric chitin administration [50,51]. Thus, chitinases and CLPs may play important roles in many pathophysiological conditions [2,38,39]. However, the contribution of chitinases and CLPs to the pathophysiology of these diseases remains to be determined.

In many studies on CLPs expression, the relative quantification using qPCR or Western blotting have been used for evaluation of CLPs levels [17,51,55,60]. Both methods involve the normalization of the expression levels of the gene of interest with those of the housekeeping genes such as GAPDH or β-actin. The relative quantification is easier to perform than the absolute quantification as the mRNA levels of the gene of interest are compared to the housekeeping genes. However, relative quantification methods fail to compare the levels of the different gene transcripts on the same scale. Although our method requires multiple steps associated with the construction of the standard DNA, it can provide gene expression data that are directly comparable between different genes.

Recent studies reported that the increased expression levels of BRP-39/YKL-40 in diseased mouse and human lungs might be the result of expression deregulation [2,38]. Furthermore, using BRP-39-deficient and YKL-40 transgenic mice, it was demonstrated that these proteins are functionally equivalent and play roles in tissue remodeling, regulation of the cell death pathway and airway obstruction [17]. Moreover, BRP-39 is induced during bacterial infection, during which it promotes bacterial clearance by controlling cell death, inflammation, and remodeling via interleukin (IL)-13 receptor α2 [60,61]. Using the quantification system described here, the CLPs mRNA levels can be compared with mammalian chitinases across mouse tissues using qPCR. This type of analysis can help to understand the biological function of CLPs, particularly in the pathophysiological studies using murine models.

## Conclusions

We established and validated a qPCR system for individual quantification of the expression of three CLPs and comparing their expression levels with those of reference genes using the same scale in mouse tissues. We found that BRP-39 was the most highly expressed CLP in the mouse lung, and its expression was comparable to that of GAPDH, a major housekeeping gene. Ym1 mRNA was also expressed at a high level in the mouse lung, whereas Ym2 mRNA was abundant in the stomach. Our results indicate that catalytically inactive BRP-39 and Ym1 are constitutively expressed in normal mouse lung.

## Abbreviations

AMCase: Acidic mammalian chitinase; BRP-39: Breast regression protein-39; Chit1: Chitotriosidase; Chi3l1: Chitinase 3-like-1; CLP: Chitinase-like protein; GAPDH: Glyceraldehyde-3-phosphate dehydrogenase; qPCR: Quantitative real-time PCR.

## Competing interests

The authors declare that they have no competing interests.

## Authors’ contributions

MO, MS, YS and FO conceived and designed the experiments. MO, YK and FO performed research. MO analyzed data. MO and FO wrote the paper. All authors contributed to revision of the manuscript and approved the final version.

## Supplementary Material

Additional file 1 Contain supporting data figures and tables and their legends. Click here for file

## References

[B1] Bussink AP Speijer D Aerts JM Boot RG Evolution of mammalian chitinase(-like) members of family 18 glycosyl hydrolases Genetics 2007 177 2 959 970 10.1534/genetics.107.075846 17720922 PMC2034658

[B2] Kawada M Hachiya Y Arihiro A Mizoguchi E Role of mammalian chitinases in inflammatory conditions Keio J Med 2007 56 1 21 27 10.2302/kjm.56.21 17392594

[B3] Hakala BE White C Recklies AD Human cartilage gp-39, a major secretory product of articular chondrocytes and synovial cells, is a mammalian member of a chitinase protein family J Biol Chem 1993 268 34 25803 25810 8245017

[B4] Rehli M Krause SW Andreesen R Molecular characterization of the gene for human cartilage gp-39 (CHI3L1), a member of the chitinase protein family and marker for late stages of macrophage differentiation Genomics 1997 43 2 221 225 10.1006/geno.1997.4778 9244440

[B5] Webb DC McKenzie AN Foster PS Expression of the Ym2 lectin-binding protein is dependent on interleukin (IL)-4 and IL-13 signal transduction: identification of a novel allergy-associated protein J Biol Chem 2001 276 45 41969 41976 10.1074/jbc.M106223200 11553626

[B6] Ward JM Yoon M Anver MR Haines DC Kudo G Gonzalez FJ Kimura S Hyalinosis and Ym1/Ym2 gene expression in the stomach and respiratory tract of 129S4/SvJae and wild-type and CYP1A2-null B6, 129 mice Am J Pathol 2001 158 1 323 332 10.1016/S0002-9440(10)63972-7 11141507 PMC1850245

[B7] Sun YJ Chang NC Hung SI Chang AC Chou CC Hsiao CD The crystal structure of a novel mammalian lectin, Ym1, suggests a saccharide binding site J Biol Chem 2001 276 20 17507 17514 10.1074/jbc.M010416200 11278670

[B8] Chang NC Hung SI Hwa KY Kato I Chen JE Liu CH Chang AC A macrophage protein, Ym1, transiently expressed during inflammation is a novel mammalian lectin J Biol Chem 2001 276 20 17497 17506 10.1074/jbc.M010417200 11297523

[B9] Jin HM Copeland NG Gilbert DJ Jenkins NA Kirkpatrick RB Rosenberg M Genetic characterization of the murine Ym1 gene and identification of a cluster of highly homologous genes Genomics 1998 54 2 316 322 10.1006/geno.1998.5593 9828134

[B10] Shackelton LM Mann DM Millis AJ Identification of a 38-kDa heparin-binding glycoprotein (gp38k) in differentiating vascular smooth muscle cells as a member of a group of proteins associated with tissue remodeling J Biol Chem 1995 270 22 13076 13083 10.1074/jbc.270.22.13076 7768902

[B11] Hu B Trinh K Figueira WF Price PA Isolation and sequence of a novel human chondrocyte protein related to mammalian members of the chitinase protein family J Biol Chem 1996 271 32 19415 19420 10.1074/jbc.271.32.19415 8702629

[B12] Arias EB Verhage HG Jaffe RC Complementary deoxyribonucleic acid cloning and molecular characterization of an estrogen-dependent human oviductal glycoprotein Biol Reprod 1994 51 4 685 694 10.1095/biolreprod51.4.685 7819450

[B13] Sendai Y Komiya H Suzuki K Onuma T Kikuchi M Hoshi H Araki Y Molecular cloning and characterization of a mouse oviduct-specific glycoprotein Biol Reprod 1995 53 2 285 294 10.1095/biolreprod53.2.285 7492680

[B14] Kzhyshkowska J Mamidi S Gratchev A Kremmer E Schmuttermaier C Krusell L Haus G Utikal J Schledzewski K Scholtze J Goerdt S Novel stabilin-1 interacting chitinase-like protein (SI-CLP) is up-regulated in alternatively activated macrophages and secreted via lysosomal pathway Blood 2006 107 8 3221 3228 10.1182/blood-2005-07-2843 16357325

[B15] Morrison BW Leder P neu and ras initiate murine mammary tumors that share genetic markers generally absent in c-myc and int-2-initiated tumors Oncogene 1994 9 12 3417 3426 7970700

[B16] Johansen JS Studies on serum YKL-40 as a biomarker in diseases with inflammation, tissue remodelling, fibroses and cancer Dan Med Bull 2006 53 2 172 209 17087877

[B17] Lee CG Hartl D Lee GR Koller B Matsuura H Da Silva CA Sohn MH Cohn L Homer RJ Kozhich AA Humbles A Kearley J Coyle A Chupp G Reed J Flavell RA Elias JA Role of breast regression protein 39 (BRP-39)/chitinase 3-like-1 in Th2 and IL-13-induced tissue responses and apoptosis J Exp Med 2009 206 5 1149 1166 10.1084/jem.20081271 19414556 PMC2715037

[B18] Henrissat B A classification of glycosyl hydrolases based on amino acid sequence similarities Biochem J 1991 280 Pt 2 309 316 1747104 10.1042/bj2800309 PMC1130547

[B19] Cantarel BL Coutinho PM Rancurel C Bernard T Lombard V Henrissat B The Carbohydrate-Active EnZymes database (CAZy): an expert resource for Glycogenomics Nucleic Acids Res 2009 37 Database issue D233 D238 18838391 10.1093/nar/gkn663 PMC2686590

[B20] Hollak CE van Weely S van Oers MH Aerts JM Marked elevation of plasma chitotriosidase activity. A novel hallmark of Gaucher disease J Clin Invest 1994 93 3 1288 1292 10.1172/JCI117084 8132768 PMC294082

[B21] Renkema GH Boot RG Muijsers AO Donker-Koopman WE Aerts JM Purification and characterization of human chitotriosidase, a novel member of the chitinase family of proteins J Biol Chem 1995 270 5 2198 2202 10.1074/jbc.270.5.2198 7836450

[B22] Boot RG Renkema GH Strijland A van Zonneveld AJ Aerts JM Cloning of a cDNA encoding chitotriosidase, a human chitinase produced by macrophages J Biol Chem 1995 270 44 26252 26256 10.1074/jbc.270.44.26252 7592832

[B23] Boot RG Blommaart EF Swart E Ghauharali-van der Vlugt K Bijl N Moe C Place A Aerts JM Identification of a novel acidic mammalian chitinase distinct from chitotriosidase J Biol Chem 2001 276 9 6770 6778 10.1074/jbc.M009886200 11085997

[B24] Watanabe T Kobori K Miyashita K Fujii T Sakai H Uchida M Tanaka H Identification of glutamic acid 204 and aspartic acid 200 in chitinase A1 of Bacillus circulans WL-12 as essential residues for chitinase activity J Biol Chem 1993 268 25 18567 18572 8103047

[B25] Sohn MH Kang MJ Matsuura H Bhandari V Chen NY Lee CG Elias JA The chitinase-like proteins breast regression protein-39 and YKL-40 regulate hyperoxia-induced acute lung injury Am J Respir Crit Care Med 2010 182 7 918 928 10.1164/rccm.200912-1793OC 20558631 PMC2970863

[B26] Letuve S Kozhich A Arouche N Grandsaigne M Reed J Dombret MC Kiener PA Aubier M Coyle AJ Pretolani M YKL-40 is elevated in patients with chronic obstructive pulmonary disease and activates alveolar macrophages J Immunol 2008 181 7 5167 5173 10.4049/jimmunol.181.7.5167 18802121

[B27] Hector A Kormann MS Mack I Latzin P Casaulta C Kieninger E Zhou Z Yildirim AO Bohla A Rieber N Kappler M Koller B Eber E Eickmeier O Zielen S Eickelberg O Griese M Mall MA Hartl D The chitinase-like protein YKL-40 modulates cystic fibrosis lung disease PLoS One 2011 6 9 e24399 10.1371/journal.pone.0024399 21949714 PMC3176766

[B28] Johansen JS Stoltenberg M Hansen M Florescu A Horslev-Petersen K Lorenzen I Price PA Serum YKL-40 concentrations in patients with rheumatoid arthritis: relation to disease activity Rheumatology (Oxford) 1999 38 7 618 626 10.1093/rheumatology/38.7.618 10461474

[B29] Bernardi D Podswiadek M Zaninotto M Punzi L Plebani M YKL-40 as a marker of joint involvement in inflammatory bowel disease Clin Chem 2003 49 10 1685 1688 10.1373/49.10.1685 14500601

[B30] Koutroubakis IE Petinaki E Dimoulios P Vardas E Roussomoustakaki M Maniatis AN Kouroumalis EA Increased serum levels of YKL-40 in patients with inflammatory bowel disease Int J Colorectal Dis 2003 18 3 254 259 12673492 10.1007/s00384-002-0446-z

[B31] Vind I Johansen JS Price PA Munkholm P Serum YKL-40, a potential new marker of disease activity in patients with inflammatory bowel disease Scand J Gastroenterol 2003 38 6 599 605 10.1080/00365520310000537 12825867

[B32] Chupp GL Lee CG Jarjour N Shim YM Holm CT He S Dziura JD Reed J Coyle AJ Kiener P Cullen M Grandsaigne M Dombret MC Aubier M Pretolani M Elias JA A chitinase-like protein in the lung and circulation of patients with severe asthma N Engl J Med 2007 357 20 2016 2027 10.1056/NEJMoa073600 18003958

[B33] Vos K Steenbakkers P Miltenburg AM Bos E van Den Heuvel MW van Hogezand RA de Vries RR Breedveld FC Boots AM Raised human cartilage glycoprotein-39 plasma levels in patients with rheumatoid arthritis and other inflammatory conditions Ann Rheum Dis 2000 59 7 544 548 10.1136/ard.59.7.544 10873965 PMC1753190

[B34] Johansen JS Moller S Price PA Bendtsen F Junge J Garbarsch C Henriksen JH Plasma YKL-40: a new potential marker of fibrosis in patients with alcoholic cirrhosis? Scand J Gastroenterol 1997 32 6 582 590 10.3109/00365529709025104 9200292

[B35] Johansen JS Cintin C Jorgensen M Kamby C Price PA Serum YKL-40: a new potential marker of prognosis and location of metastases of patients with recurrent breast cancer Eur J Cancer 1995 31A 9 1437 1442 7577068 10.1016/0959-8049(95)00196-p

[B36] Cintin C Johansen JS Christensen IJ Price PA Sorensen S Nielsen HJ Serum YKL-40 and colorectal cancer Br J Cancer 1999 79 9-10 1494 1499 10188896 10.1038/sj.bjc.6690238 PMC2362720

[B37] Hung SI Chang AC Kato I Chang NC Transient expression of Ym1, a heparin-binding lectin, during developmental hematopoiesis and inflammation J Leukoc Biol 2002 72 1 72 82 12101265

[B38] Lee CG Da Silva CA Dela Cruz CS Ahangari F Ma B Kang MJ He CH Takyar S Elias JA Role of chitin and chitinase/chitinase-like proteins in inflammation, tissue remodeling, and injury Annu Rev Physiol 2011 73 479 501 10.1146/annurev-physiol-012110-142250 21054166 PMC3864643

[B39] Bueter CL Specht CA Levitz SM Innate sensing of chitin and chitosan PLoS Pathog 2013 9 1 e1003080 10.1371/journal.ppat.1003080 23326227 PMC3542151

[B40] Ohno M Tsuda K Sakaguchi M Sugahara Y Oyama F Chitinase mRNA levels by quantitative PCR using the single standard DNA: acidic mammalian chitinase is a major transcript in the mouse stomach PLoS One 2012 7 11 e50381 10.1371/journal.pone.0050381 23185612 PMC3503932

[B41] Ohno M Togashi Y Tsuda K Okawa K Kamaya M Sakaguchi M Sugahara Y Oyama F Quantification of chitinase mRNA levels in human and mouse tissues by real-time PCR: species-specific expression of acidic mammalian chitinase in stomach tissues PLoS One 2013 8 6 e67399 10.1371/journal.pone.0067399 23826286 PMC3694897

[B42] Kouadjo KE Nishida Y Cadrin-Girard JF Yoshioka M St-Amand J Housekeeping and tissue-specific genes in mouse tissues BMC Genomics 2007 8 127 10.1186/1471-2164-8-127 17519037 PMC1888706

[B43] Dabek J Wilczok J Kulach A Gasior Z Altered transcriptional activity of gene encoding GAPDH in peripheral blood mononuclear cells from patients with cardiac syndrome X - an important part in pathology of microvascular angina? Arch Med Sci 2010 6 5 709 712 22419929 10.5114/aoms.2010.17085 PMC3298339

[B44] Zainuddin A Chua KH Abdul Rahim N Makpol S Effect of experimental treatment on GAPDH mRNA expression as a housekeeping gene in human diploid fibroblasts BMC Mol Biol 2010 11 59 10.1186/1471-2199-11-59 20707929 PMC2930638

[B45] Bustin SA Benes V Garson JA Hellemans J Huggett J Kubista M Mueller R Nolan T Pfaffl MW Shipley GL Vandesompele J Wittwer CT The MIQE guidelines: minimum information for publication of quantitative real-time PCR experiments Clin Chem 2009 55 4 611 622 10.1373/clinchem.2008.112797 19246619

[B46] Bustin SA Beaulieu JF Huggett J Jaggi R Kibenge FS Olsvik PA Penning LC Toegel S MIQE precis: Practical implementation of minimum standard guidelines for fluorescence-based quantitative real-time PCR experiments BMC Mol Biol 2010 11 74 10.1186/1471-2199-11-74 20858237 PMC2955025

[B47] Nygard AB Jorgensen CB Cirera S Fredholm M Selection of reference genes for gene expression studies in pig tissues using SYBR green qPCR BMC Mol Biol 2007 8 67 10.1186/1471-2199-8-67 17697375 PMC2000887

[B48] Kageyama T Pepsinogens, progastricsins, and prochymosins: structure, function, evolution, and development Cell Mol Life Sci 2002 59 2 288 306 10.1007/s00018-002-8423-9 11915945 PMC11146132

[B49] O’Connell J The basics of RT-PCR. Some practical considerations Methods Mol Biol 2002 193 19 25 12325508 10.1385/1-59259-283-X:019

[B50] Zhu Z Zheng T Homer RJ Kim YK Chen NY Cohn L Hamid Q Elias JA Acidic mammalian chitinase in asthmatic Th2 inflammation and IL-13 pathway activation Science 2004 304 5677 1678 1682 10.1126/science.1095336 15192232

[B51] Reese TA Liang HE Tager AM Luster AD Van Rooijen N Voehringer D Locksley RM Chitin induces accumulation in tissue of innate immune cells associated with allergy Nature 2007 447 7140 92 96 10.1038/nature05746 17450126 PMC2527589

[B52] Seibold MA Donnelly S Solon M Innes A Woodruff PG Boot RG Burchard EG Fahy JV Chitotriosidase is the primary active chitinase in the human lung and is modulated by genotype and smoking habit J Allergy Clin Immunol 2008 122 5 944 950 e943 10.1016/j.jaci.2008.08.023 18845328 PMC2666777

[B53] Boot RG Bussink AP Verhoek M de Boer PA Moorman AF Aerts JM Marked differences in tissue-specific expression of chitinases in mouse and man J Histochem Cytochem 2005 53 10 1283 1292 10.1369/jhc.4A6547.2005 15923370

[B54] van Eijk M van Roomen CP Renkema GH Bussink AP Andrews L Blommaart EF Sugar A Verhoeven AJ Boot RG Aerts JM Characterization of human phagocyte-derived chitotriosidase, a component of innate immunity Int Immunol 2005 17 11 1505 1512 10.1093/intimm/dxh328 16214810

[B55] Qureshi AM Hannigan A Campbell D Nixon C Wilson JB Chitinase-like proteins are autoantigens in a model of inflammation-promoted incipient neoplasia Genes Cancer 2011 2 1 74 87 10.1177/1947601911402681 21779482 PMC3111005

[B56] Kashimura A Okawa K Ishikawa K Kida Y Iwabuchi K Matsushima Y Sakaguchi M Sugahara Y Oyama F Protein A-mouse acidic mammalian chitinase-V5-His expressed in periplasmic space of Escherichia coli possesses chitinase functions comparable to CHO-expressed protein PLoS One 2013 8 11 e78669 10.1371/journal.pone.0078669 24244337 PMC3823863

[B57] Zheng T Rabach M Chen NY Rabach L Hu X Elias JA Zhu Z Molecular cloning and functional characterization of mouse chitotriosidase Gene 2005 357 1 37 46 10.1016/j.gene.2005.05.006 16005164

[B58] Letuve S Kozhich A Humbles A Brewah Y Dombret MC Grandsaigne M Adle H Kolbeck R Aubier M Coyle AJ Pretolani M Lung chitinolytic activity and chitotriosidase are elevated in chronic obstructive pulmonary disease and contribute to lung inflammation Am J Pathol 2010 176 2 638 649 10.2353/ajpath.2010.090455 20042671 PMC2808072

[B59] Watabe-Rudolph M Song Z Lausser L Schnack C Begus-Nahrmann Y Scheithauer MO Rettinger G Otto M Tumani H Thal DR Attems J Jellinger KA Kestler HA von Arnim CA Rudolph KL Chitinase enzyme activity in CSF is a powerful biomarker of Alzheimer disease Neurology 2012 78 8 569 577 10.1212/WNL.0b013e318247caa1 22323746

[B60] He CH Lee CG Dela Cruz CS Lee CM Zhou Y Ahangari F Ma B Herzog EL Rosenberg SA Li Y Nour AM Parikh CR Schmidt I Modis Y Cantley L Elias JA Chitinase 3-like 1 regulates cellular and tissue responses via IL-13 receptor alpha2 Cell Rep 2013 4 4 830 841 10.1016/j.celrep.2013.07.032 23972995 PMC3988532

[B61] Dela Cruz CS Liu W He CH Jacoby A Gornitzky A Ma B Flavell R Lee CG Elias JA Chitinase 3-like-1 promotes Streptococcus pneumoniae killing and augments host tolerance to lung antibacterial responses Cell Host Microbe 2012 12 1 34 46 10.1016/j.chom.2012.05.017 22817986 PMC3613130

