# Supplementary material for: Establishment of a quantitative PCR system for discriminating chitinase-like proteins: catalytically inactive breast regression protein-39 and Ym1 are constitutive genes in mouse lung
Source: BMC Mol Biol. 2014 Oct 8;15:23. doi: 10.1186/1471-2199-15-23 (PMC4195342; doi:10.1186/1471-2199-15-23)
Supplement: Additional file 1 — Contain supporting data figures and tables and their legends. [file 1471-2199-15-23-S1.pdf]

Figure S1 Nucleotide sequence of the mouse Refs/CLPs standard DNA. The mouse Refs/CLPs standard DNA (1,597 nucleotides long) contained eight cDNA fragments (shown in different colors) that covered the PCR target regions (shown in bold and underlined) and 9-146 nucleotides of the flanking regions and contained the BglII, XhoI, PstI, NotI and EcoRI restriction sites (shown in bold and italics).

Mw 986,684.8

GTGGATTCTGTGCCGACAAAGCAGATGGCCTCTACCCTGTGGCAGATGACAGAAATGCT
TTTTGGCAGTGCATCAATGGAATCACATACCAGCAGCATTGTCAAGCAGGGCTTGTTT**T**
TGATACCAGCTGTAATTGCTGCAACTGGCCA***AGATCT***TGGCAACCAGGCCTCTGGCTGG
TGCTCCTCCTCTGGC**TGCCAAGGCATTGTAGACACA**GGCACCTCTCTGCTCGTCATGCC
TGCCCAGTACCTG**AATGAACTTCTGCAGACCATAGGAG**CCCAGGAAGGAGAGTATGGAC
AGTATTTTGT***CTCGAG***TGGACTTGGATGACTTCAAGGGTTCCTTCTGCAACCAGGGCCC
GTACCCTCTCATCCGGACACTA**CGGCAGGAACTAAATCTTCCAT**CCGAGACTCCAAGGA
GCCCAGAACAGATA**ATACCTGAGCCACGCCCA**TCTTCTATGCCAGAGCAGGGACCCAGC
CCAGGGCTA***CTGCAG***GAGCTGAACGGGAAGCTCACTGGCATGGCCTTCCGTGTTCCTAC
CCCCAA**TGTGTCCGTCGTGGATCTGA**CGTGCCGCCTGGAGAAACCTGCCAAGTATGATG
ACA**TCAAGAAGGTGGTGAAGCAGG**CATCTGAGGGCCCACTGAAGGGCATCTTGGGCTAC
ACT***GCGGCCGC***CGAGCAGGAGATGGCCACTGCCGCATCCTCTTCCTCCCTGGAGAAGAG
CTATGAGCTGCCTG**ACGGCCAGGTCATCACTATTG**GCAACGAGCGGTTCCGATGCCCTG
AGGC**TCTTTTCCAGCCTTCCTTCTTG**GGTATGGAATCCTGTGGCATCCATGAAACTACA
TTCAATTCCATCATGAAGTGTGACGTTGACATCCGTAAAGACCTCTATGCCAACACAGT
GCTGTCTGGTGGTACCACCATGTACCCA***GAATTC***ATTCCTTCGTCAATATAACTTTGAT
GGCCTCAACCTGGACTGGCAGTACCCTGGGTCTCGAGGAAGCCCTCCTAAGGACAAACA
TCTCTTCAGTGT**TCTGGTGAAGGAAATGCGTAAA**GCTTTTGAGGAAGAATCTGTG**GAGA**
AAGACATTCCAAGGCTGCTACTCACTTCCACAGGAGCAGGAATCATTGACGTAATCAAG
TCTGGGTACAAGATCCCTGAACTGTCTCAGTCTCTTGACTATATTCAGG***AGATCT***ACAA
AGGAGGT**CCAGCCAGGCAGAGAGAAAC**TCCTGCTCAGCGCAGCTT**TGTCAGCAGGAAAG**
GTGGCCATTGACACTGGCTATGACATCGCCCAGATAGCCCAACACCTGGATTTTATCAA
TCTCATGACCTACGATTTCCATGGAGTCTGGCGCCAAATCACAGGCCA***CTCGAG***GAAGC
CCTCCTAAGGACAAACATCTCTTCAGTGT**TCTGGTGCAGGAAATGCGTAAA**GCTTTTGA
GGAAGAATCCA**CTTTGAACCACATTCCAAGGCTGC**TACTCACTTCCACAGGAGCTGGAT
TCATTGACGTAATCAAGTCTGGGTACAAGATCCCTGAACTGTCTCAGTCTCTCGACTAT
ATTCAGGTCATGACATATGATCTCCATGATCCTAAGAATGGCTACACTGGAGAGAGCTC
CATG

Figure S2 Nucleotide sequence of the mouse Refs/CLPs standard DNA with pGEM-T Easy. The mouse Refs/CLPs standard DNA with pGEM-T Easy (4,629 nucleotides long) consisted of pGEM-T Easy (shown in black) and eight cDNA fragments (shown in different colors) that covered the PCR target regions (shown in bold and underlined) and 9-146 nucleotides of the flanking regions and contained the BglII, SalI, XhoI, NotI and EcoRI restriction sites (shown in bold and italics).

Mw 2,860,159.6

CATG***AGATCT***ACAAAGGAGGT**CCAGCCAGGCAGAGAGAAAC**TCCTGCTCAGCGCAGCTT
TGTCAGCAGGAAAGGTGGCCATTGACACTGGCTATGACATCGCCCAGATAGCCCAACAC
CTGGATTTTATCAATCTCATGACCTACGATTTCCATGGAGTCTGGCGCCAAATCACAGG
CCA***CTCGAG***GAAGCCCTCCTAAGGACAAACATCTCTTCAGTGT**TCTGGTGCAGGAAATG**
CGTAAAGCTTTTGAGGAAGAATCCA**CTTTGAACCACATTCCAAGGCTGC**TACTCACTTC
CACAGGAGCTGGATTCATTGACGTAATCAAGTCTGGGTACAAGATCCCTGAACTGTCTC
AGTCTCTCGACTATATTCAGGTCATGACATATGATCTCCATGATCCTAAGAATGGCTAC
ACTGGAGAGAGCTCCATGAATCGAATTCCCGCGGCCGCCATGGCGGCCGGGAGCATGCG
ACGTCGGGCCCAATTCGCCCTATAGTGAGTCGTATTACAATTCACTGGCCGTCGTTTTA
CAACGTCGTGACTGGGAAAACCCTGGCGTTACCCAACTTAATCGCCTTGCAGCACATCC
CCCTTTCGCCAGCTGGCGTAATAGCGAAGAGGCCCGCACCGATCGCCCTTCCCAACAGT
TGCGCAGCCTGAATGGCGAATGGACGCGCCCTGTAGCGGCGCATTAAGCGCGGCGGGTG
TGGTGGTTACGCGCAGCGTGACCGCTACACTTGCCAGCGCCCTAGCGCCCGCTCCTTTC
GCTTTCTTCCCTTCCTTTCTCGCCACGTTCGCCGGCTTTCCCCGTCAAGCTCTAAATCG
GGGGCTCCCTTTAGGGTTCCGATTTAGTGCTTTACGGCACCTCGACCCCAAAAAACTTG
ATTAGGGTGATGGTTCACGTAGTGGGCCATCGCCCTGATAGACGGTTTTTCGCCCTTTG
ACGTTGGAGTCCACGTTCTTTAATAGTGGACTCTTGTTCCAAACTGGAACAACACTCAA
CCCTATCTCGGTCTATTCTTTTGATTTATAAGGGATTTTGCCGATTTCGGCCTATTGGT
TAAAAAATGAGCTGATTTAACAAAAATTTAACGCGAATTTTAACAAAATATTAACGCTT
ACAATTTCCTGATGCGGTATTTTCTCCTTACGCATCTGTGCGGTATTTCACACCGCATC
AGGTGGCACTTTTCGGGGAAATGTGCGCGGAACCCCTATTTGTTTATTTTTCTAAATAC
ATTCAAATATGTATCCGCTCATGAGACAATAACCCTGATAAATGCTTCAATAATATTGA
AAAAGGAAGAGTATGAGTATTCAACATTTCCGTGTCGCCCTTATTCCCTTTTTTGCGGC
ATTTTGCCTTCCTGTTTTTGCTCACCCAGAAACGCTGGTGAAAGTAAAAGATGCTGAAG
ATCAGTTGGGTGCACGAGTGGGTTACATCGAACTGGATCTCAACAGCGGTAAGATCCTT
GAGAGTTTTCGCCCCGAAGAACGTTTTCCAATGATGAGCACTTTTAAAGTTCTGCTATG
TGGCGCGGTATTATCCCGTATTGACGCCGGGCAAGAGCAACTCGGTCGCCGCATACACT
ATTCTCAGAATGACTTGGTTGAGTACTCACCAGTCACAGAAAAGCATCTTACGGATGGC
ATGACAGTAAGAGAATTATGCAGTGCTGCCATAACCATGAGTGATAACACTGCGGCCAA

CTTACTTCTGACAACGATCGGAGGACCGAAGGAGCTAACCGCTTTTTTGCACAACATGG
GGGATCATGTAACTCGCCTTGATCGTTGGGAACCGGAGCTGAATGAAGCCATACCAAAC
GACGAGCGTGACACCACGATGCCTGTAGCAATGGCAACAACGTTGCGCAAACTATTAAC
TGGCGAACTACTTACTCTAGCTTCCCGGCAACAATTAATAGACTGGATGGAGGCGGATA
AAGTTGCAGGACCACTTCTGCGCTCGGCCCTTCCGGCTGGCTGGTTTATTGCTGATAAA
TCTGGAGCCGGTGAGCGTGGGTCTCGCGGTATCATTGCAGCACTGGGGCCAGATGGTAA
GCCCTCCCGTATCGTAGTTATCTACACGACGGGGAGTCAGGCAACTATGGATGAACGAA
ATAGACAGATCGCTGAGATAGGTGCCTCACTGATTAAGCATTGGTAACTGTCAGACCAA
GTTTACTCATATATACTTTAGATTGATTTAAAACTTCATTTTTAATTTAAAAGGATCTA
GGTGAAGATCCTTTTTGATAATCTCATGACCAAAATCCCTTAACGTGAGTTTTCGTTCC
ACTGAGCGTCAGACCCCGTAGAAAAGATCAAAGGATCTTCTTGAGATCCTTTTTTTCTG
CGCGTAATCTGCTGCTTGCAAACAAAAAAACCACCGCTACCAGCGGTGGTTTGTTTGCC
GGATCAAGAGCTACCAACTCTTTTTCCGAAGGTAACTGGCTTCAGCAGAGCGCAGATAC
CAAATACTGTTCTTCTAGTGTAGCCGTAGTTAGGCCACCACTTCAAGAACTCTGTAGCA
CCGCCTACATACCTCGCTCTGCTAATCCTGTTACCAGTGGCTGCTGCCAGTGGCGATAA
GTCGTGTCTTACCGGGTTGGACTCAAGACGATAGTTACCGGATAAGGCGCAGCGGTCGG
GCTGAACGGGGGGTTCGTGCACACAGCCCAGCTTGGAGCGAACGACCTACACCGAACTG
AGATACCTACAGCGTGAGCTATGAGAAAGCGCCACGCTTCCCGAAGGGAGAAAGGCGGA
CAGGTATCCGGTAAGCGGCAGGGTCGGAACAGGAGAGCGCACGAGGGAGCTTCCAGGGG
GAAACGCCTGGTATCTTTATAGTCCTGTCGGGTTTCGCCACCTCTGACTTGAGCGTCGA
TTTTTGTGATGCTCGTCAGGGGGGCGGAGCCTATGGAAAAACGCCAGCAACGCGGCCTT
TTTACGGTTCCTGGCCTTTTGCTGGCCTTTTGCTCACATGTTCTTTCCTGCGTTATCCC
CTGATTCTGTGGATAACCGTATTACCGCCTTTGAGTGAGCTGATACCGCTCGCCGCAGC
CGAACGACCGAGCGCAGCGAGTCAGTGAGCGAGGAAGCGGAAGAGCGCCCAATACGCAA
ACCGCCTCTCCCCGCGCGTTGGCCGATTCATTAATGCAGCTGGCACGACAGGTTTCCCG
ACTGGAAAGCGGGCAGTGAGCGCAACGCAATTAATGTGAGTTAGCTCACTCATTAGGCA
CCCCAGGCTTTACACTTTATGCTTCCGGCTCGTATGTTGTGTGGAATTGTGAGCGGATA
ACAATTTCACACAGGAAACAGCTATGACCATGATTACGCCAAGCTATTTAGGTGACACT
ATAGAATACTCAAGCTATGCATCCAACGCGTTGGGAGCTCTCCCATATGGTCGACCTGC
AGGCGGCCGCGAATTCACTAGTGATTTGTGGATTCTGTGCCGACAAAGCAGATGGCCTC
TACCCTGTGGCAGATGACAGAAATGCT**TTTTGGCAGTGCATCAATGG**AATCACATACCA
GCAGCATTGTCAAGCAGGGCTTGTTT**TTGATACCAGCTGTAATTGCTGC**AACTGGCCA***A***
GATCTTGGCAACCAGGCCTCTGGCTGGTGCTCCTCCTCTGGC**TGCCAAGGCATTGTAGA**
CACAGGCACCTCTCTGCTCGTCATGCCTGCCCAGTACCTG**AATGAACTTCTGCAGACCA**
TAGGAGCCCAGGAAGGAGAGTATGGACAGTATTTTGT***CTCGAG***TGGACTTGGATGACTT
CAAGGGTTCCTTCTGCAACCAGGGCCCGTACCCTCTCATCCGGACACTA**CGGCAGGAAC**

TAAATCTTCCATCCGAGACTCCAAGGAGCCCAGAACAGATA**ATACCTGAGCCACGCCCA**
TCTTCTATGCCAGAGCAGGGACCCAGCCCAGGGCTA***CTGCAG***GAGCTGAACGGGAAGCT
CACTGGCATGGCCTTCCGTGTTCCTACCCCCAA**TGTGTCCGTCGTGGATCTGA**CGTGCC
GCCTGGAGAAACCTGCCAAGTATGATGACA**TCAAGAAGGTGGTGAAGCAGG**CATCTGAG
GGCCCACTGAAGGGCATCTTGGGCTACACT***GCGGCCGC***CGAGCAGGAGATGGCCACTGC
CGCATCCTCTTCCTCCCTGGAGAAGAGCTATGAGCTGCCTG**ACGGCCAGGTCATCACTA**
TTGGCAACGAGCGGTTCCGATGCCCTGAGGC**TCTTTTCCAGCCTTCCTTCTTG**GGTATG
GAATCCTGTGGCATCCATGAAACTACATTCAATTCCATCATGAAGTGTGACGTTGACAT
CCGTAAAGACCTCTATGCCAACACAGTGCTGTCTGGTGGTACCACCATGTACCCA***GAAT***
TCATTCCTTCGTCAATATAACTTTGATGGCCTCAACCTGGACTGGCAGTACCCTGGGTC
TCGAGGAAGCCCTCCTAAGGACAAACATCTCTTCAGTGT**TCTGGTGAAGGAAATGCGTA**
AAGCTTTTGAGGAAGAATCTGTG**GAGAAAGACATTCCAAGGCTGC**TACTCACTTCCACA
GGAGCAGGAATCATTGACGTAATCAAGTCTGGGTACAAGATCCCTGAACTGTCTCAGTC
TCTTGACTATATTCAGG***AGATCT***GTCA

Figure S3 Nucleotide sequence and calculated molecular weight of BRP-39, Ym1 and Ym2 entire coding cDNAs.

BRP-39

MW 767,970.2

CATGGGATCCGTGGAGCCTAAGGAAGAGGCCCTGACTAGGAAGCTGGGTACTAGGAGAA
GCCATCATGCACACCTCTACTGAAGCCAGGATGGGCATGAGGGCGGCACTGACAGGCTT
TGCGGTCCTGATGCTGCTCCAGAGCTGCTCTGCGTACAAGCTGGTCTGCTACTTCACCA
GCTGGTCCCAGTACCGGGAAGGCGTTGGAAGCTTCTTACCAGACGCCATCCAACCTTTC
CTGTGCACCCACATCATCTACAGCTTTGCCAACATCAGCAGCGACAACATGCTTAGCAC
ATGGGAGTGGAATGACGAGTCGAACTATGACAAGCTGAATAAACTGAAGACCAGAAACA
CCAACCTGAAGACCCTCCTGTCTGTTGGAGGGTGGAAATTTGGCGAAAAAGATTTTCC
GAGATTGCCTCCAACACTGAGAGACGCACTGCTTTCGTCCGGTCGGTAGCCCCGTTCCT
GCGTTCTTATGGCTTTGATGGGCTGGATCTCGCCTGGCTCTACCCTCGCTTAAGAGACA
AGCAGTATTTCTCCACCCTGATCAAGGAACTGAATGCGGAATTCACAAAGGAGGTCCAG
CCAGGCAGAGAGAAACTCCTGCTCAGCGCAGCTTTGTCAGCAGGAAAGGTGGCCATTGA
CACTGGCTATGACATCGCCCAGATAGCCCAACACCTGGATTTTATCAATCTCATGACCT
ACGATTTCCATGGAGTCTGGCGCCAAATCACAGGCCATCACAGCCCCCTCTTCCAAGGC
CAGAAGGACACTAGGTTTGACAGATACAGCAATGTGAACTATGCCGTGCAGTACATGAT
ACGTCTGGGAGCCCAGGCCAGCAAGCTACTGATGGGCATCCCCACCTTTGGGAAGAGCT
TCACTCTGGCATCTTCTGAAAATCAGTTGGGAGCTCCAATCTCAGGGGAAGGATTACCA

GGCCGGTTCACCAAGGAGGCAGGGACCCTGGCCTACTACGAGATATGCGACTTCCTCAA
AGGAGCTGAAGTACATCGACTCTCCAACGAGAAGGTTCCCTTCGCTACCAAGGGCAACC
AGTGGGTGGGGTATGAGGACAAGGAGAGTGTCAAAAACAAGGTTGGGTTCCTGAAGGAG
AAGAAGCTGGCAGGAGCCATGGTGTGGGCACTGGATTTGGATGATTTCCAGGGCACCTG
TCAGCCGAAGGAATTCTTCCCGCTCACCAACGCCATCAAGGATGCCCTGGCTGCTCGAG
GTCA

Ym1
MW 760,470.4
CATGGAATTCAATCCTGAAGACACCATGGCCAAGCTCATTCTTGTCACAGGTCTGGCAA
TTCTTCTGAACGTACAGCTGGGATCTTCCTACCAGCTGATGTGCTACTATACCAGTTGG
GCTAAGGACAGGCCAATAGAAGGGAGTTTCAAACCTGGTAATATTGACCCCTGCCTGTG
TACTCACCTGATCTATGCCTTTGCTGGAATGCAGAATAATGAGATCACTTACACACATG
AGCAAGACTTGCGTGACTATGAAGCATTGAATGGTCTGAAAGACAAGAACACTGAGCTA
AAAACTCTCCTGGCCATTGGAGGATGGAAGTTTGGACCTGCCCCGTTCAGTGCCATGGT
CTCTACTCCTCAGAACCGTCAGATATTCATTCAGTCAGTTATCAGATTCCTTCGTCAAT
ATAACTTTGATGGCCTCAACCTGGACTGGCAGTACCCTGGGTCTCGAGGAAGCCCTCCT
AAGGACAAACATCTCTTCAGTGTTCTGGTGAAGGAAATGCGTAAAGCTTTTGAGGAAGA
ATCTGTGGAGAAAGACATTCCAAGGCTGCTACTCACTTCCACAGGAGCAGGAATCATTG
ACGTAATCAAGTCTGGGTACAAGATCCCTGAACTGTCTCAGTCTCTTGACTATATTCAG
GTCATGACATATGATCTCCATGATCCTAAGGATGGCTACACTGGAGAAAATAGTCCCCT
CTATAAATCTCCATATGACATTGGAAAGAGTGCTGATCTCAATGTGGATTCAATCATTT
CCTACTGGAAGGACCATGGAGCAGCTTCTGAGAAGCTCATTGTGGGATTTCCAGCATAT
GGGCATACCTTTATCCTGAGTGACCCTTCTAAGACTGGAATTGGTGCCCCTACAATTAG
TACTGGCCCACCAGGAAAGTACACAGATGAATCAGGACTCCTGGCTTACTATGAGGTTT
GTACATTTCTGAATGAAGGAGCCACTGAGGTCTGGGATGCCCCCCAGGAAGTACCCTAT
GCCTATCAGGGTAATGAGTGGGTTGGTTATGACAATGTCAGGAGCTTCAAGTTGAAGGC
TCAGTGGCTCAAGGACAACAATTTAGGAGGTGCCGTGGTCTGGCCCCTGGACATGGATG
ACTTCAGTGGTTCTTTCTGTCACCAGAGACATTTCCCTCTGACATCTACTTTAAAGGGA
GATCTCAATATACACAGTGCAAGTTGCAAGGGCCCTTATGCGGCCGCTCGA

Ym2
MW 767,879.2
CATGGAATTCAATCCTGAAGACACCATGGCCAAGCTCATTCTTGTCACAGGTCTGGCAA
TTCTTCTGAATGTACAGCTGGGATCTTCCTACCAGCTGATGTGCTACTATACCAGCTGG
GCTAAGGACAGGCCAACAGAAGGGAGTTTCAAACCTGGTAATATTGACCCCTGCCTGTG

TACTCACCTGATCTATGCCTTTGCTGGGATGAAGAATAATGAGATCACTTACTTAAGTG
AGCAAGACTTGCGTGACTATGAAGCATTAAATGGTCTGAAAGACAGGAACACTGAGCTA
AAAACTCTCCTGGCCATTGGAGGATGGAAGTTTGGACCTGCCCCGTTCAGTTCCATGGT
CTCTACTCCTCAGAACCGTCAGACATTCATTAAGTCAGTTATCAGATTCCTTCGTCAAT
ATAACTTTGATGGCCTCAACCTGGACTGGCAGTACCCTGGGTCTCGAGGAAGCCCTCCT
AAGGACAAACATCTCTTCAGTGTTCTGGTGCAGGAAATGCGTAAAGCTTTTGAGGAAGA
ATCCACTTTGAACCACATTCCAAGGCTGCTACTCACTTCCACAGGAGCTGGATTCATTG
ACGTAATCAAGTCTGGGTACAAGATCCCTGAACTGTCTCAGTCTCTCGACTATATTCAG
GTCATGACATATGATCTCCATGATCCTAAGAATGGCTACACTGGAGAAAATAGTCCCCT
CTATAAATCTCCATATGACATTGGAAAGAGTGCTGATCTCAATGTGGATTCAATTATTA
CCTACTGGAAGGACCATGGAGCAGCTTCTGAGAAGCTCATTGTGGGATTTCCAGCATAT
GGTCATACCTTTATCCTGAGTGACCCTTCTAAGAATGGAATAGGTGACCCTACTGTTAG
TGCTGGACCACCAGGAAAGTACACAAATGAACAAGGACTCCTGGCTTACTTTGAGATTT
GTACATTTCTGAATGAAGGAGCCACTGAGATCTTTGATGCCACCCAGGAAGTACCCTAT
GCCTATCTGGGTAATGAGTGGGTTGGTTATGACAATGTCAGGAGCTTCAAGTTGAAGGC
TCAGTGGCTCAAGGACAACAATTTAGGAGGTGCCGTGGTCTGGCCCCTGGACATGGATG
ACTTCAGTGGTTCTTTCTGTCACCAGGGACGTTTCCCTCTGACAACTACTTTAAAGAGA
GATCTGAATGTACACAGTGCAAGTTGCAAGGCCTCTTATCGAGGGGAGCTTGCGGCCGC
TCGA

Figure S4. Comparison of the nucleotide sequence of the Ym1 with that of Ym2.
The nucleotide sequences of the Ym1 and Ym2 primers are shown underlined in pink.

Identities: 1423/1511(94%)

```
Ym1  29    AATCCTGAAGACACCATGGCCAAGCTCATTCTTGTCACAGGTCTGGCAATTCTTCTGAAC  88
           ||||||||||||||||||||||||||||||||||||||||||||||||||||||||||| 
Ym2  1     AATCCTGAAGACACCATGGCCAAGCTCATTCTTGTCACAGGTCTGGCAATTCTTCTGAAT  60

Ym1  89    GTACAGCTGGGATCTTCCTACCAGCTGATGTGCTACTATACCAGTTGGGCTAAGGACAGG  148
           |||||||||||||||||||||||||||||||||||||||||||| |||||||||||||||
Ym2  61    GTACAGCTGGGATCTTCCTACCAGCTGATGTGCTACTATACCAGCTGGGCTAAGGACAGG  120

Ym1  149   CCAATAGAAGGGAGTTTCAAACCTGGTAATATTGACCCCTGCCTGTGTACTCACCTGATC  208
           |||| |||||||||||||||||||||||||||||||||||||||||||||||||||||||
Ym2  121   CCAACAGAAGGGAGTTTCAAACCTGGTAATATTGACCCCTGCCTGTGTACTCACCTGATC  180

Ym1  209   TATGCCTTTGCTGGAATGCAGAATAATGAGATCACTTACACACATGAGCAAGACTTGCGT  268
           |||||||||||||| ||| ||||||||||||||||||||  |  ||||||||||||||||
Ym2  181   TATGCCTTTGCTGGGATGAAGAATAATGAGATCACTTACTTAAGTGAGCAAGACTTGCGT  240

Ym1  269   GACTATGAAGCATTGAATGGTCTGAAAGACAAGAACACTGAGCTAAAAACTCTCCTGGCC  328
           |||||||||||||| |||||||||||||||| ||||||||||||||||||||||||||||
Ym2  241   GACTATGAAGCATTAAATGGTCTGAAAGACAGGAACACTGAGCTAAAAACTCTCCTGGCC  300
```

```
Ym1  329   ATTGGAGGATGGAAGTTTGGACCTGCCCCGTTCAGTGCCATGGTCTCTACTCCTCAGAAC  388
           |||||||||||||||||||||||||||||||||||| |||||||||||||||||||||||
Ym2  301   ATTGGAGGATGGAAGTTTGGACCTGCCCCGTTCAGTTCCATGGTCTCTACTCCTCAGAAC  360

Ym1  389   CGTCAGATATTCATTCAGTCAGTTATCAGATTCCTTCGTCAATATAACTTTGATGGCCTC  448
           ||||||| ||||||| ||||||||||||||||||||||||||||||||||||||||||||
Ym2  361   CGTCAGACATTCATTAAGTCAGTTATCAGATTCCTTCGTCAATATAACTTTGATGGCCTC  420

Ym1  449   AACCTGGACTGGCAGTACCCTGGGTCTCGAGGAAGCCCTCCTAAGGACAAACATCTCTTC  508
           ||||||||||||||||||||||||||||||||||||||||||||||||||||||||||||
Ym2  421   AACCTGGACTGGCAGTACCCTGGGTCTCGAGGAAGCCCTCCTAAGGACAAACATCTCTTC  480

Ym1  509   AGTGTTCTGGTGAAGGAAATGCGTAAAGCTTTTGAGGAAGAATCTGTGGAGAAAGACATT  568
           |||||||||||| |||||||||||||||||||||||||||||||      |||  |||||
Ym2  481   AGTGTTCTGGTGCAGGAAATGCGTAAAGCTTTTGAGGAAGAATCCACTTTGAACCACATT  540

Ym1  569   CCAAGGCTGCTACTCACTTCCACAGGAGCAGGAATCATTGACGTAATCAAGTCTGGGTAC  628
           ||||||||||||||||||||||||||||| ||| ||||||||||||||||||||||||||
Ym2  541   CCAAGGCTGCTACTCACTTCCACAGGAGCTGGATTCATTGACGTAATCAAGTCTGGGTAC  600

Ym1  629   AAGATCCCTGAACTGTCTCAGTCTCTTGACTATATTCAGGTCATGACATATGATCTCCAT  688
           |||||||||||||||||||||||||| |||||||||||||||||||||||||||||||||
Ym2  601   AAGATCCCTGAACTGTCTCAGTCTCTCGACTATATTCAGGTCATGACATATGATCTCCAT  660

Ym1  689   GATCCTAAGGATGGCTACACTGGAGAAAATAGTCCCCTCTATAAATCTCCATATGACATT  748
           ||||||||| ||||||||||||||||||||||||||||||||||||||||||||||||||
Ym2  661   GATCCTAAGAATGGCTACACTGGAGAAAATAGTCCCCTCTATAAATCTCCATATGACATT  720

Ym1  749   GGAAAGAGTGCTGATCTCAATGTGGATTCAATCATTTCCTACTGGAAGGACCATGGAGCA  808
           |||||||||||||||||||||||||||||||| ||| |||||||||||||||||||||||
Ym2  721   GGAAAGAGTGCTGATCTCAATGTGGATTCAATTATTACCTACTGGAAGGACCATGGAGCA  780

Ym1  809   GCTTCTGAGAAGCTCATTGTGGGATTTCCAGCATATGGGCATACCTTTATCCTGAGTGAC  868
           |||||||||||||||||||||||||||||||||||||| |||||||||||||||||||||
Ym2  781   GCTTCTGAGAAGCTCATTGTGGGATTTCCAGCATATGGTCATACCTTTATCCTGAGTGAC  840

Ym1  869   CCTTCTAAGACTGGAATTGGTGCCCCTACAATTAGTACTGGCCCACCAGGAAAGTACACA  928
           |||||||||| |||||| |||| ||||||  ||||| |||| ||||||||||||||||||
Ym2  841   CCTTCTAAGAATGGAATAGGTGACCCTACTGTTAGTGCTGGACCACCAGGAAAGTACACA  900

Ym1  929   GATGAATCAGGACTCCTGGCTTACTATGAGGTTTGTACATTTCTGAATGAAGGAGCCACT  988
            |||||  ||||||||||||||||| |||| |||||||||||||||||||||||||||||
Ym2  901   AATGAACAAGGACTCCTGGCTTACTTTGAGATTTGTACATTTCTGAATGAAGGAGCCACT  960

Ym1  989   GAGGTCTGGGATGCCCCCCAGGAAGTACCCTATGCCTATCAGGGTAATGAGTGGGTTGGT  1048
           ||| |||  |||||| |||||||||||||||||||||||| |||||||||||||||||||
Ym2  961   GAGATCTTTGATGCCACCCAGGAAGTACCCTATGCCTATCTGGGTAATGAGTGGGTTGGT  1020

Ym1  1049  TATGACAATGTCAGGAGCTTCAAGTTGAAGGCTCAGTGGCTCAAGGACAACAATTTAGGA  1108
           ||||||||||||||||||||||||||||||||||||||||||||||||||||||||||||
Ym2  1021  TATGACAATGTCAGGAGCTTCAAGTTGAAGGCTCAGTGGCTCAAGGACAACAATTTAGGA  1080

Ym1  1109  GGTGCCGTGGTCTGGCCCCTGGACATGGATGACTTCAGTGGTTCTTTCTGTCACCAGAGA  1168
           ||||||||||||||||||||||||||||||||||||||||||||||||||||||||| ||
Ym2  1081  GGTGCCGTGGTCTGGCCCCTGGACATGGATGACTTCAGTGGTTCTTTCTGTCACCAGGGA  1140

Ym1  1169  CATTTCCCTCTGACATCTACTTTAAAGGGAGATCTCAATATACACAGTGCAAGTTGCAAG  1228
           | ||||||||||||| ||||||||||| ||||||| ||| ||||||||||||||||||||
```

```
Ym2  1141  CGTTTCCCTCTGACAACTACTTTAAAGAGAGATCTGAATGTACACAGTGCAAGTTGCAAG  1200

Ym1  1229  GGCCCTTATTGAGAGGAGCTTTACACAATGATTTGTCCTTGAAACTCTCAGAATAAGATC  1288
           | | ||||| ||| ||||||||| |||||||||| | ||||||||||||||||||||| |
Ym2  1201  GCCTCTTATCGAGGGGAGCTTTAGACAATGATTTCTACTTGAAACTCTCAGAATAAGAGC  1260

Ym1  1289  AAGTTCAACGGTTTTTCCACAGTGCATTCTGCATCATGCTTCCATGGAGAATAATAGAAA  1348
           || |||||||||||||||||  || ||||||||||||||||||||||||||||||||||
Ym2  1261  AACTTCAACGGTTTTTCCACGATGGATTCTGCATCATGCTTCCATGGAGAATAATAGAAC  1320

Ym1  1349  TAAGTCATGAACTTTCCTAAATTGAATCCCAGAGTAGTACTAAGATGGATGTCTTGTCTG  1408
           ||||||| || ||||||| |||||||||||||||||| ||||||||  ||||||||||||
Ym2  1321  TAAGTCACGATCTTTCCTGAATTGAATCCCAGAGTAGAACTAAGATATATGTCTTGTCTG  1380

Ym1  1409  CTGTACCAGCTGGGAAGAAAC-AAAAAATGCTCTTCATCTGTCAGCTTTGGCTAAGCTCT  1467
           || ||||||| |  ||||||| |||||||  |||||||||||||||||||||||||||||
Ym2  1381  CTATACCAGCAGAAAAGAAACAAAAAAATTATCTTCATCTGTCAGCTTTGGCTAAGCTCT  1440

Ym1  1468  GAACATCTTTTGCTTCCTGTAAAACCACCATGCTTGTTTCTTGCTCTCACAATAAATTCC  1527
           |||||| |||||||||||||||||||||| | |||||||||| || ||| ||||||||||
Ym2  1441  GAACAT-TTTTGCTTCCTGTAAAACCACCTTCCTTGTTTCTTCCTTTCATAATAAATTCC  1499

Ym1  1528  ACATTCATAGC  1538
           |  |||| |||
Ym2  1500  AAGTTCAAAGC  1510
```

Table S1. The nucleotide sequences of the real-time PCR primers.

```
Ym1_Fw: TCTGGTGAAGGAAATGCGTAAA
Ym1_Rv: GCAGCCTTGGAATGTCTTTCTC

Ym2_Fw: TCTGGTGCAGGAAATGCGTAAA
Ym2_Rv: GCAGCCTTGGAATGTGGTTCAAAG

BRP-39_Fw: CCAGCCAGGCAGAGAGAAAC
BRP-39_Rv: GCCACCTTTCCTGCTGACA
```

Table S2 Forward and reverse primers used to construct the standard DNAs.

EcoRI_Ym1_Fw: **CATG**GAATTCATTCCTTCGTCAATATAACTTTGAT

BglII_Ym1_Rv: **TGAC**AGATCTCCTGAATATAGTCAAGAGACTGAGA

BglII_BRP-39_Fw: **CATG**AGATCTACAAAGGAGGTCCAGCCAGGCAGAG

XhoI_BRP-39_Rv: **TGAC**CTCGAGTGGCCTGTGATTTGGCGCCAGACTC

XhoI_Ym2_Fw: GTACCCTGGGTCTCGAGGAAGCCCTC
Ym2_Rv: CATGGAGCTCTCTCCAGTGTAGCCATTCTTAGGAT

Quant_Mouse_AMCase_Fw: GTGGATTCTGTGCCGACAAAGCAGATGGCC

Table S3 Primers for PCR amplification of the entire coding cDNAs.

Entire_BRP-39_Fw: CATGGGATCCGTGGAGCCTAAGGAAGAGGCCCTGA
Entire_BRP-39_Rv: TGACCTCGAGCAGCCAGGGCATCCTTGATGGCGTT

Entire_Ym1_Fw: CATGGAATTCAATCCTGAAGACACCATGGCCAAGC
Entire_Ym1_RV: TCGAGCGGCCGCATAAGGGCCCTTGCAACTTGCAC

Entire_Ym2_FW: CATGGAATTCAATCCTGAAGACACCATGGCCAAGC
Entire_Ym2_RV: TCGAGCGGCCGCAAGCTCCCCTCGATAAGAGGCCT
